# Increasing Number of Individuals Receiving Hepatitis B nucleos(t)ide Analogs Therapy in Germany, 2008–2019

**DOI:** 10.3389/fpubh.2021.667253

**Published:** 2021-05-21

**Authors:** Anna Maisa, Christian Kollan, Matthias an der Heiden, Florian van Bömmel, Markus Cornberg, Stefan Mauss, Heiner Wedemeyer, Daniel Schmidt, Sandra Dudareva

**Affiliations:** ^1^Department of Infectious Disease Epidemiology, Robert Koch Institute, Berlin, Germany; ^2^Division of Hepatology, Department of Medicine II, Leipzig University Medical Center, Leipzig, Germany; ^3^Department of Gastroenterology, Hepatology and Endocrinology, Hannover Medical School, Hannover, Germany; ^4^Center for HIV and Hepatogastroenterology, Düsseldorf, Germany

**Keywords:** hepatitis B, nucleos(t)ide therapy, therapy costs, treatment guidelines, treatment gap, hepatitis elimination

## Abstract

**Background:** Germany is a low prevalence country for hepatitis B virus (HBV) infection with higher prevalence in vulnerable groups. The number of treated chronic hepatitis B (CHB) patients is unknown. We aimed to determine the number of CHB patients treated with nucleos(t)ide analogs (NUCs), the treatment costs within the statutory health insurance (SHI) in Germany and per patient per month.

**Methods:** Data on pharmacy bills of NUCs to patients with SHI between 2008 and 2019 were purchased from Insight Health™ and described. Negative binomial regression was used for trend analysis.

**Results:** Number of patients increased between 2008 and 2019 (4.9% per year) with little changes in treatment options. Overall prescription costs were increasing (6.7% per year on average) until the introduction of tenofovir and entecavir generics in 2017 after which costs decreased by 31% in 2019. Average therapy costs peaked at 498 Euro per patient per month in 2016 and decreased to 214 Euro in 2019. Prescriptions changed from 30 to 90 pills per pack over time. HBV therapy was prescribed to 97% by three medical specialist groups, mainly specialists in internal medicine (63%), followed by hospital-based outpatient clinics (20%) and general practitioners (15%). Contrary to guideline recommendation, adefovir was still prescribed after 2011 for 1–5% of patients albeit with decreasing tendency. Prescriptions per 100,000 inhabitants were highest in Berlin and Hamburg.

**Conclusion:** Our data shows, that the number of treated CHB patients increased steadily, while NUC therapy costs decreased. We recommend continued testing and treatment for those eligible to prevent advanced liver disease and possibly decrease further transmission of HBV.

## Introduction

Despite the availability of antivirals and highly effective vaccines, chronic hepatitis B (CHB) infections are still prevalent globally. In 2015, 257–270 million people worldwide were living with hepatitis B virus (HBV) and 686,000 deaths related to liver cirrhosis and hepatocellular carcinoma were attributed to HBV infection (1). Only 9% of infected individuals worldwide were aware of their infection and 8% of those, were receiving care (1).

Although the European Union (EU) and European Economic Area (EEA) are low prevalence regions, there is wide variation among countries and 4.7 million people are estimated to be chronically infected with HBV (2).

Germany is a low prevalence country with 0.3% of hepatitis B surface antigen (HBsAg) prevalence in the general population and between 0.2 and 4.5% in vulnerable groups (3, 4). Based on this, it has been estimated that 227,000 [95% confidence interval (CI) 174,000–287,000] adults and 25,000 (95% CI 14,000–43,000) children were living with HBV infection in Germany in 2013 (Kremer et al. Number of people living with hepatitis B and C in Germany, 2013. Manuscript in preparation). However, the number of individuals treated for CHB is unknown.

Elimination of viral hepatitis is based on prevention of infection, and there is an ongoing need for diagnosis and treatment of chronic hepatitis B in addition to population-based vaccination to be able to reach the WHO elimination goal for viral hepatitis in 2030 (5).

There were no changes in the availability of medical treatment options for CHB since 2008, except for the introduction of the new drug tenofovir alafenamide (TAF) in April 2017 (6). According to German HBV treatment guidelines, the NUCs available for CHB treatment included lamivudine (3TC), entecavir (ETV), telbivudine (LdT), and tenofovir disoproxil fumarate (TDF) (6). The use of adefovir (ADV) was discouraged due to toxicities (6). The generics for 3TC, ETV and TDF were introduced in March 2012, May 2017 and August 2017, respectively.

### Aim

The aim of our analysis was to estimate the number of people receiving NUC treatment for HBV based on pharmacy billing data, and to describe the costs associated with NUC therapy in Germany from 2008 to 2019 over time and per patient treated per month.

## Methods

### Data Source

Prescription data on NUCs for HBV therapy from 2008 until 2019 were purchased from Insight Health™. Monthly data was collected from billing centers that processed all reimbursed prescriptions from pharmacies. Insight Health™ claimed a coverage of >99% within the statutory health insurance (SHI) prescription market. The SHI covers up to 88% of the German population (7). The data on pharmacy sales is a stand-alone non-person-specific database and cannot be linked to further health insurance data. There is currently no accessible national data source for the SHI system, which would allow validation of prescriptions according to diagnoses. Furthermore, there is considerable miscoding within SHI data regarding diagnoses. The data source used here have a special legal status in Germany and can generally be purchased with a delay of 3 months for all SHI-insured individuals.

For each NUC the defined daily dose per patient according to treatment guidelines is one pill a day (6). Prescriptions included pack sizes of 30 pills (P30, one-month supply), 60 pills (P60, 2 months' supply) and 90 pills (P90, 3 months' supply). For the purpose of this study, prescriptions were divided into 30 pill monthly units (MU) and shown as monthly frequencies representing the approximate number of individuals treated. Annual frequencies represent prescriptions per year, and are not equivalent to number of patients treated per year. Data collected included substance name, pack size, and number of prescriptions including respective costs, as well as information on the prescriber and location of prescription reimbursement. The pharmacy price per drug at the respective time point was divided by MU to obtain monthly costs.

### Analysis

We describe the monthly frequencies and proportions of patients and prescriptions over time stratified by drug. Monthly prescription costs per patient were calculated by dividing the cumulative monthly costs by the number of patients per month. Annual overall costs consist of the total cost of all prescriptions per year. Data is presented as counts and proportions. Rates of prescriptions are displayed per 100,000 inhabitants.

We estimated annual trends in prescription rates using negative binomial regression, presented as incidence rate ratios and percentage change. Linear and quadratic function of time was used to find best fit for trend. For cost analysis, likelihood ratio test was used to determine better fit of trend data. Trend numbers calculated represent numbers of prescriptions in January for each year based on monthly prescriptions.

All analyses were carried out using Microsoft Excel 2010 and STATA® (version 15). QGIS 3.12 was used for creating maps and shapefiles were obtained from Esri DE Open Data © GeoBasis-DE / BKG 2018 (8).

### Ethical Statement

Ethical approval and informed consent were not required as our study used anonymised secondary data from pharmacies.

## Results

**Figure 1:**
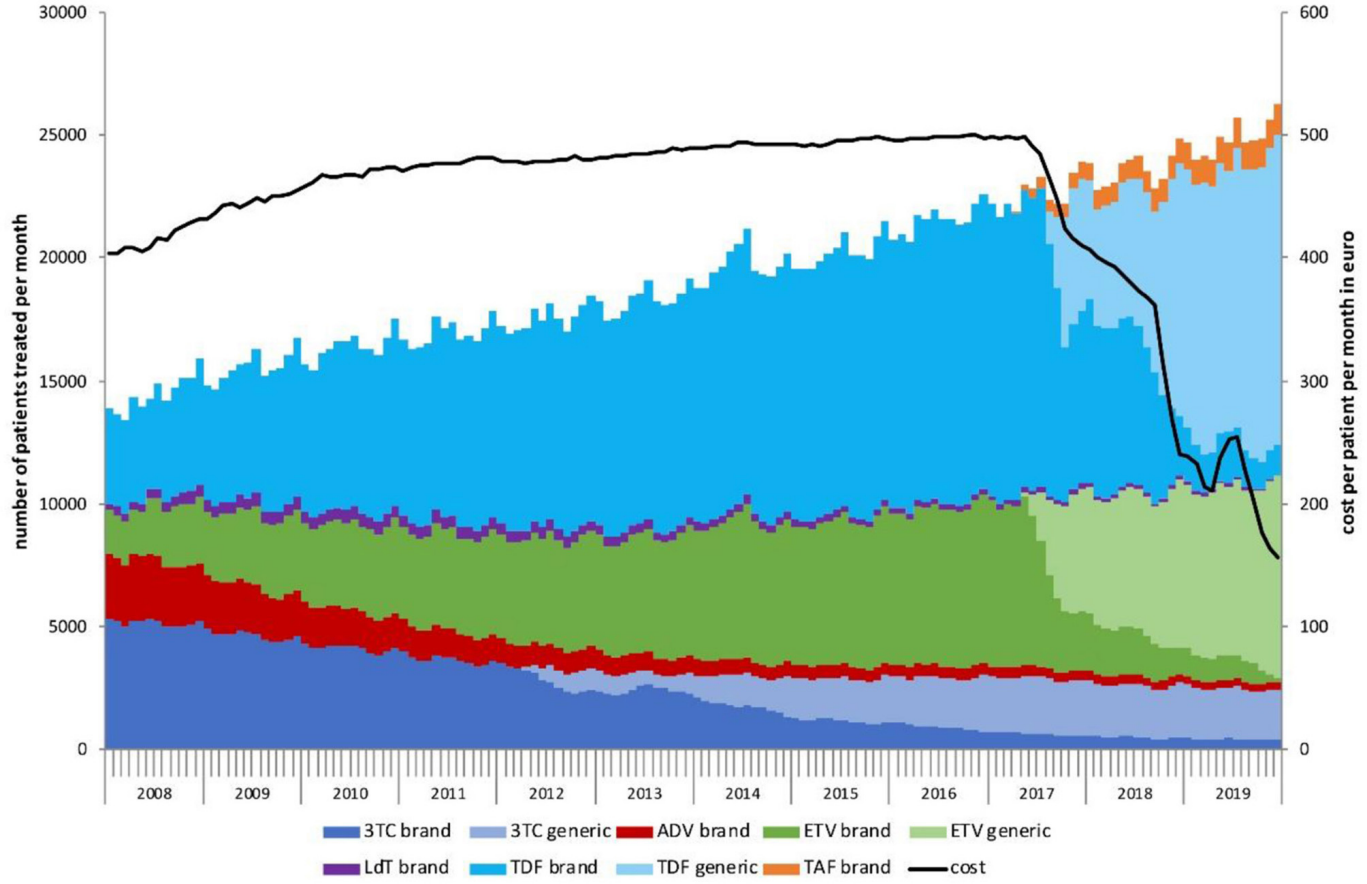
Number of chronic hepatitis B patients treated per month by drug, 2008–2019. The black line shows the cost per patient per month in Euro.

The overall number of prescriptions in our study period was 1,150,779, which was equivalent to 2,780,783 MU. The corresponding average number of patients being treated was 14,453 per month in 2008 increasing to 24,868 per month in 2019 (Figure 1).

In 2008, 36% of prescriptions were for 3TC, 29% for TDF, 18% for ADV, 15% for ETV and 2.8% for LdT (Supplementary Table 1). By 2019, 9.9% of prescriptions were for 3TC (including 8.2% for the generic drug), 1.3% for ADV, 31% for ETV (including 28% for the generic drug), 0.5% for LdT, 53% prescriptions were for TDF (including 46% for the generic drug) and 4.5% for TAF. ADV continued to be prescribed after 2011 for patients although with reduced frequency (5.2% in 2012 to 1.3% in 2019).

From 2008 to 2019, the average number of treated patients increased by 4.9% per year and overall, by 70% (Table 1). During this period, we observed a substantial change in the treatment landscape. The monthly frequency of 3TC prescriptions is steadily decreasing and overall, by 51% in 2019 compared to 2008. ADV showed the largest decline in prescriptions, with 88% decrease between 2008 and 2019, followed by LdT with a decrease of 74%. Prescriptions of ETV increased in 2019 compared to 2008 by 252%, followed by TDF with an increase of 203%. TAF was introduced in 2017 as a new therapy option and prescriptions have increased by 42% in 2019 compared to 2018. This change in prescriptions over time is consistent with a slight increase in average costs.

**Table 1:** Annual number and MU percentage change from prescribed NUC drugs for HBV therapy, 2008–2019.

	**Total MU**		**3TC**		**ADV**		**ETV**		**LdT**		**TDF**		**TAF**	
**Year**	***n***	**% change**	***n***	**% change**	***n***	**% change**	***n***	**% change**	***n***	**% change**	***n***	**% change**	***n***	**% change**
2008	14,326		5,363		2,933		2,139				4,111			
2009	15,022	4.9	4,763	−11	2,190	−25	2,580	21	525		4,980	21		
2010	15,752	4.9	4,275	−10	1,668	−24	3,067	19	506	−3.6	5,924	19		
2011	16,520	4.9	3,878	−9.3	1,295	−22	3,593	17	477	−5.7	6,920	17		
2012	17,328	4.9	3,555	−8.3	1,025	−21	4,149	15	440	−7.7	7,936	15		
2013	18,177	4.9	3,295	−7.3	827	−19	4,720	14	398	−9.7	8,938	13		
2014	19,071	4.9	3,086	−6.3	680	−18	5,293	12	351	−12	9,884	11		
2015	20,010	4.9	2,921	−5.3	571	−16	5,849	11	304	−14	10,733	8.6		
2016	20,998	4.9	2,794	−4.3	488	−14	6,370	8.9	257	−15	11,444	6.6		
2017	22,038	5.0	2,702	−3.3	425	−13	6,836	7.3	213	−17	11,982	4.7		
2018	23,132	5.0	2,641	−2.3	378	−11	7,231	5.8	172	−19	12,318	2.8	719	
2019	24,283	5.0	2,608	−1.2	342	−9.4	7,538	4.2	137	−21	12,435	0.9	1018	42

*MU, monthly units representing number of individuals treated; 3TC, lamivudine; ADV, adefovir; ETV, entecavir; LdT, telbivudine; TDF, tenofovir disoproxil fumarate; TAF, tenofovir alafenamide*.

The total cost of prescriptions over the study period rose from 74 million Euro in 2008, to a peak of 129 million Euro in 2016, and then dropped to 62 million Euro in 2019 (Supplementary Figure 2; Supplementary Table 3). In 2009, we observed an increase of 9.2% compared to 2008, which was slowing down to 4.2% in 2017 compared to 2016, and plummeting by 31% in 2019 compared to 2018. The average cost per patient per month increased from 415 Euro in 2008 to 498 Euro in 2016 and dropped to 214 Euro in 2019 (Figure 1).

The majority of prescriptions (97%) were issued by specialists in internal medicine (63%; including hepatologists and gastroenterologists), hospital-based outpatient clinics (20%), and general practitioners (15%). The yearly average proportion of prescribed NUCs by these three specialist groups is consistent with the proportions provided (Supplementary Table 1) and according to the proportion of prescriptions issued by each specialist group (data not shown).

**Figure 2:**
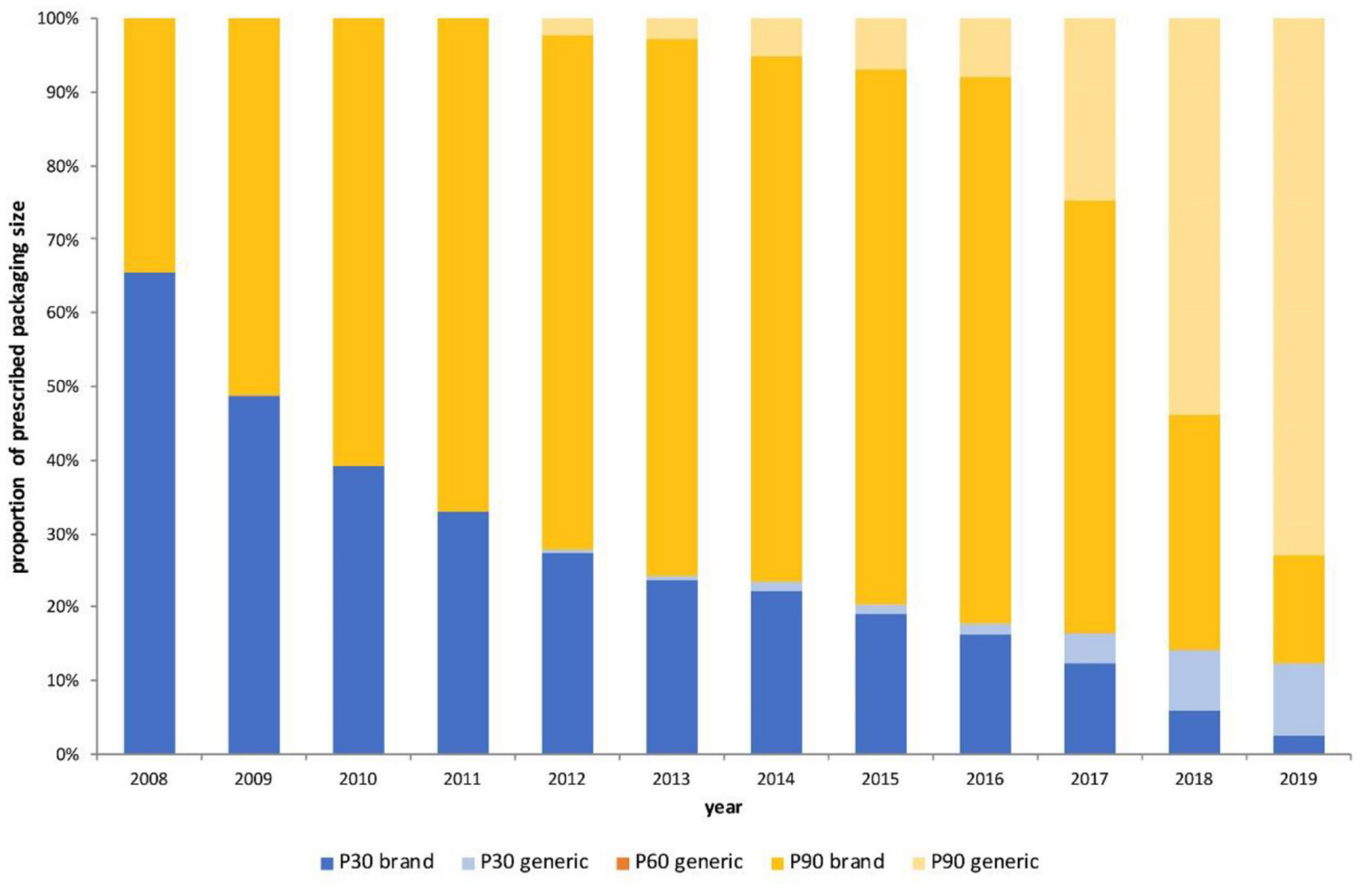
Proportion of prescriptions issued per year by prescription size, 2008–2019. P30–30 pills per prescription; P60–60 pills per prescription; P90–90 pills per prescription.

Regarding size of pack prescribed, we observed a shift from 30-pill packs per prescription (P30) to 90-pill packs (P90) (Figure 2). Frequency of P30 prescribing declined by 80% from 2008 to 2019, whereas prescribing of P90 increased by 164%. Generic prescriptions were available as P30 and P90. In 2019, the P90 to P30 ratio for the brand-name drug was 5.7:1 and for the generic 7.4:1. The 60-pill pack (P60) was very rarely prescribed (<0.2%).

**Figure 3:**
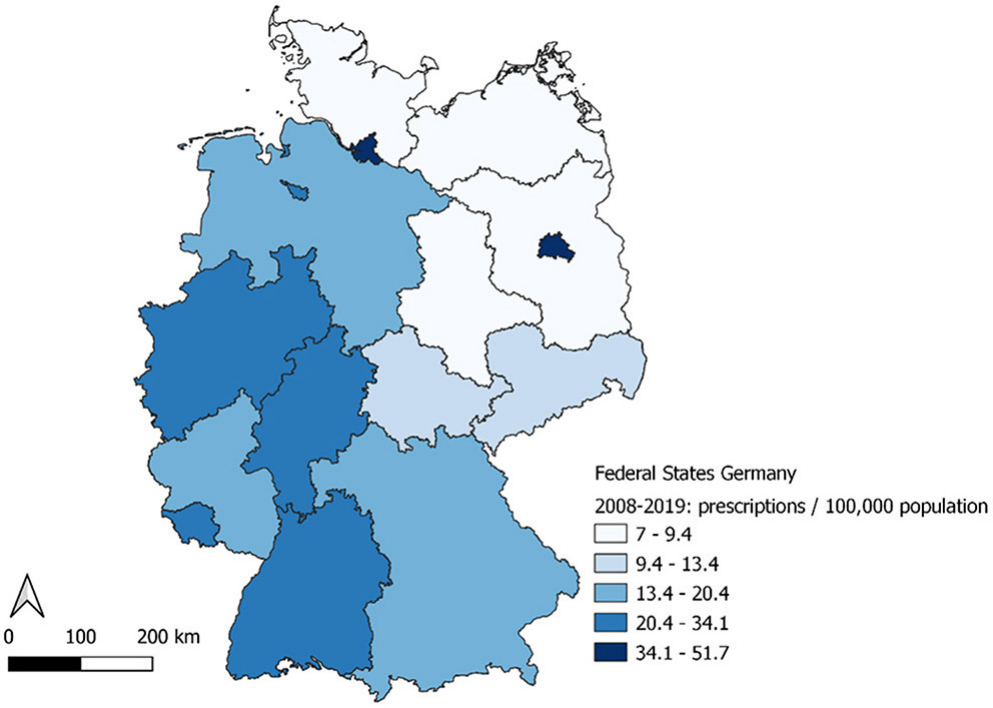
Prescriptions per 100,000 population by Federal State, Germany, 2008–2019.

In terms of geographical distribution, prescription rates in the 16 Germany Federal States ranged from 6.5 to 52 per 100,000 inhabitants (Figure 3). Highest prescriptions rates were observed in Berlin (52/100,000) and Hamburg (49/100,000); with lowest rates in Brandenburg (6.5/100,000), Mecklenburg-Western Pomerania (8.4/100,000), Saxony-Anhalt (8.8/100,000) and Schleswig-Holstein (8.9/100,000).

Over the study period, the prescription rate increase ranged from an average of 0.59% per year in Berlin to an average of 7.97% per year in Bremen. Average increase was over 5% per year in 11 of the Federal States (Supplementary Table 4).

## Discussion

Our analysis shows that from 2008, the number of CHB treated individuals with NUCs increased continuously over time with a therapy mostly according to current guidelines (6). Treatment costs decreased substantially after 2017 with the introduction of generics.

The data suggests long-term treatment of CHB patients and the growing tendency of individuals on therapy is reflected by the steady notification of new acute HBV cases in Germany (9). In 2013, an estimate suggested 252,000 adults (95% CI: 190,000–334,000) in the general population in Germany were HBsAg positive (9). Our analysis shows that 87,656 prescriptions of NUCs were issued in 2013. Due to the long-term therapy, this does not reflect the actual number of treated patients and the increase in treated patients reflect the cohort effect of treated patients who survive for a long time. Instead, we estimate an average of 18,283 patients on therapy per month in 2013, representing a proportion of 8.1% of the estimate of HBsAg positive adults and reflecting a gap between individuals living with HBV in Germany in 2013 and the ones on treatment. In addition, our data shows a steady increase of individuals on CHB treatment despite a potential influx of migrants during 2015 (10). This steady increase of prescriptions may be due to an increased availability of NUC therapy and increased elimination efforts of viral hepatitis. However, our estimates were based on NUC therapy as only a limited number of individuals is receiving PEG-IFN (pegylated interferon) for hepatitis B (data not shown). A recent study from the USA described the proportion of CHB patients with antiviral treatment claims in 2016 as 18% and the proportion of CHB patients treated with PEG-IFN as 2.8% (11). This emphasizes that a gap would remain, even though we underestimate the number of treated individuals in our dataset. In addition, a large proportion of HBsAg positive patients does not require treatment and the proportion of the estimated HBsAg positive adults in 2013 eligible for treatment is unknown (12).

Treatment with TDF is likely to have been overestimated in our dataset, as this drug is also used in Human Immunodeficiency Virus (HIV) therapy. However, combination therapy with TDF for HIV patients was introduced after 2005 and single therapy of TDF in HIV should be uncommon from then on. Overestimation, especially in the first few years of our dataset, would ultimately lead to a steeper increase of HBV patients on therapy at the earlier time points.

Observed HBV treatment is in accordance with treatment guidelines with the exception of the prescription of ADV, which is no longer recommended due to nephrotoxicity and availability of alternative drugs since 2011 (6). However, there was a progressive reduction in use, with 5.2% receiving ADV in 2012, declining to 1.3% in 2019. Still, continued education on available treatment options is needed to prevent prescriptions drugs with lower efficacy and higher risk for side effects.

Our data also shows that HBV NUC therapy was mostly prescribed by a limited number of physician groups, despite the option to obtain a prescription from all medical doctors in Germany. This underlines that medical encounters and prescriptions for HBV are most likely to occur in specialized care. The recent recommendation in Germany for HBV screening to be a part of regular health checks for those aged 35 and over, may be beneficial for the detection of previously unknown cases and may help scaling up the number of patients on treatment (13).

Reasons for the observed shift in prescribed pack size may include anticipation of longer treatment and efforts to increase convenience for the patient and to reduce the administrative and/or labor costs of the pharmacy and/or the doctor. An alternative explanation is that P30 is prescribed for treatment initiation or to bridge the absence of a medical specialist.

Prescription rates were highest in the federal city states of Berlin and Hamburg. Correspondingly, the highest incidence rates of acute HBV infections are reported in these cities (9). This effect would be probably also seen in other large cities in Germany, which is not noticeable on federal state level as it is for the federal city states. One reason for the higher incidence rates in Berlin and Hamburg as well as in the highly industrialized areas in West Germany is probably the higher rate of people from countries with higher HBV incidence. Additionally, fewer specialized centers or medical specialists for HBV might be located in mostly rural areas in Eastern Germany.

Mother-to-child transmission is the driving force of new HBV infections in high prevalence countries, and as a result, most CHB patients with a migration background are adults, who were infected in childhood (14). In Germany, HBV childhood vaccination was introduced in 1995 and current CHB patients have most likely not benefited from childhood vaccination yet. This is supported by the fact that the 500–700 new acute HBV cases notified in Germany every year since 2008 have a median age of 43 (9).

Our data also suggests treatment costs seem not to be a limiting factor as patient numbers grow steadily. In addition, there was no substantial change in overall prescription numbers after the introduction of the two generic drugs and/or the drop in treatment costs in 2017. However, it is not known if the number of diagnosed patients is a limiting factor considering the treatment gap. The slight increase of average cost per patient per month between 2008 and 2016 can be explained by a change in combination of drugs prescribed over time.

The substantial change in costs per patient per month after 2017 would most likely decrease the economic burden of CHB treatment in Germany. However, the cost of treatment is likely negligible compared to CHB long-term health effects. Challenges remain to identify individuals that are infected, and eligible for treatment, as wells as ensuring adherence to treatment, especially in vulnerable or hard to reach groups.

### Limitations

Our analysis is limited due to a potential underestimation of the data, which only includes individuals in Germany with SHI. However, in 2019, up to 88% of the German population were covered by SHI (7). Moreover, individuals from risk and vulnerable groups, for example, intravenous drug users (IDU), migrants and asylum seekers, and homeless people may, at some point, not be covered by any form of health insurance (15, 16). Furthermore, our data on pharmacy sales is not linked to health insurance data and it is not possible to validate prescription data vs. diagnosis. In addition, we did not consider patients on PEG-IFN treatment in our analysis as they are difficult to distinguish from patients with diagnoses other than CHB. However, a recent study from the USA describes the proportion of CHB patients treated with PEG IFN as 2.8% (11). With our dataset, we cannot account for possible co-infections, which were described as 1% for HCV/HBV and 2.3% for HIV/HBV by others (11) and might be negligible since HBV in HIV/HBV co-infections will be covered by HIV combination therapy. Our data also does not account for treatment interruptions, combination therapies, or adherence to treatment, but considering that the majority of patients will receive the standard of care monotherapy and remain on long term NUC treatment.

## Conclusion

Our findings suggest that the number of CHB patients on treatment according to German guidelines is increasing, which leads to an interruption of transmission chains and the prevention of progression to more severe disease. However, there is still a large gap between patients who should potentially receive antiviral therapy and patients receiving treatment. There is an ongoing need for diagnosis and treatment of HBV infection in order to reach the WHO elimination goal for viral hepatitis in 2030. To further increase the number of CHB patients on treatment, we recommend continuing to test and treat.

## Data Availability Statement

The data analyzed in this study is subject to the following licenses/restrictions: Secondary data were purchased from Insight Health™ and license for further distribution applies. Aggregated data available by reasonable request to the corresponding author. Requests to access these datasets should be directed to Sandra Dudareva, DudarevaS@rki.de.

## Author Contributions

AM, CK, DS, and SD designed the study. AM, CK, and MH performed data analysis. AM drafted the manuscript. All authors interpreted the data and critically reviewed the manuscript.

## Conflict of Interest

MC declares to having received payment or consultancy fees from Gilead Sciences, Janssen-Cilag, Spring Bank Pharmaceuticals, GlaxoSmithKline and Roche on the subject of hepatitis B. MC department or institution has received funding from Roche and Gilead Sciences for research projects as well as from Gilead Sciences and Janssen-Cilag for carrying out clinical studies. SM declares to having received payment or consultancy fees from Janssen-Cilag. FB declares to having received payment or consultancy fees from Gilead Sciences, Janssen-Cilag, Abbvie, Arbutus, Springbank and Roche and BMS; payment to cover cost of participation in a conference or educational event, travel or accommodation expenses related to the topic from Gilead Sciences, Janssen-Cilag and Roche; research funding from Gilead Sciences, Janssen-Cilag and Roche; and funding for carrying out clinical studies from Gilead Sciences. HW declares to having received payment for lectures or consultancy fees from Abbott, Abbvie, Altimmune, Biotest, BMS, BTG, Dicerna, Gilead, Janssen, Merck/MSD, MYR GmbH, Novartis, Roche and Siemens; research funding from Abbvie, Biotest, BMS, Gilead, Merck/MSD, Novartis and Roche; and funding for carrying out clinical studies from Abbvie, Altimmune, BMS, Gilead, Janssen, Merck/MSD, MYR GmbH, Novartis and Transgene. The remaining authors declare that the research was conducted in the absence of any commercial or financial relationships that could be construed as a potential conflict of interest.

## References

[1] World Health Organization. Global Hepatitis Report, 2017. (2017). p. 62.

[2] European Centre for Disease Prevention and Control. Systematic review on hepatitis B and C prevalence in the EU/EEA. Ecdc. (2016). Available online at: https://www.ecdc.europa.eu/sites/default/files/media/en/publications/Publications/systematic-review-hepatitis-B-C-prevalence.pdf (accessed February 12, 2021).

[3] Sperle I Steffen G Leendertz SA Sarma N Beermann S Thamm R. Prevalence of hepatitis B, C, and D in Germany: results from a scoping review. Front Public Health. (2020) 8:424. 10.3389/fpubh.2020.00424 33014960 PMC7493659

[4] Poethko-Müller C Zimmermann R Hamouda O Faber M Stark K Ross RS. Die Seroepidemiologie der Hepatitis A, B und C in Deutschland. Vol. 56. Robert Koch-Institut, Epidemiologie und Gesundheitsberichterstattung. Robert Koch-Institut, Infektionsepidemiologie (2013).

[5] World Health Organization. Global Health Sector Strategy on Viral Hepatitis 2016-2021. Towards Ending Viral Hepatitis. Geneva: World Health Organization (2016).

[6] Cornberg M Protzer U Petersen J Wedemeyer H Berg T Jilg W. Aktualisierung der S 3-Leitlinie zur Prophylaxe, Diagnostik und Therapie der Hepatitis-B-Virusinfektion. Z Gastroenterol. (2011) 49:871–930. 10.1055/s-0031-1273462 21748700

[7] Bundesministerium für Gesundheit. Daten des Gesundheitswesens 2009. (2009).

[8] Esri DE Open Data © GeoBasis-DE / BKG (2018). Available online at: https://opendata-esri-de.opendata.arcgis.com/datasets/b8d0cc7735774bed8e6df1c5410394a4_0 (accessed February 12, 2021).

[9] Dudareva S Kremer K Harder T Maisa A Bremer V Zimmermann R. Virushepatitis B und D im Jahr (2019). Epid Bull. (2020) 30/31:17–31. 10.25646/7025

[10] von Laer A Diercke M An der Heiden M Altmann D Zimmermann R Dudareva S. Implications of a change in case definition and screening of asylum seekers for hepatitis B surveillance in Germany in 2015 and (2016). Epidemiol Infect. (2020) 148:e36. 10.1017/S0950268820000242 32089143 PMC7058648

[11] Harris AM Osinubi A Nelson NP Thompson WW. The hepatitis B care cascade using administrative claims data, 2016. Am J Manag Care. (2020) 26:331–8. 10.37765/ajmc.2020.44069 32835460

[12] zu Siederdissen CH Cornberg M. The role of HBsAg levels in the current management of chronic HBV infection. Ann Gastroenterol. (2014) 27:105–12. 24733569 PMC3982624

[13] Pressemitteilung: Screening auf Hepatitis B und C neuer Bestandteil des Gesundheits-Check-ups. Berlin, 20. November (2020). Available online at: https://www.g-ba.de/presse/pressemitteilungen/912/ (accessed November 29, 2020).

[14] World Health Organization. Action Plan for the Health Sector Response to Viral Hepatitis in the WHO European Region. Denkmark: WHO Regional Office for Europe, (2016).

[15] Linke C Heintze C Holzinger F. 'Managing scarcity'-a qualitative study on volunteer-based healthcare for chronically ill, uninsured migrants in Berlin, Germany. BMJ Open. (2019) 9:1–9. 10.1136/bmjopen-2018-025018 30904858 PMC6475233

[16] Kaduszkiewicz H Bochon B Van Den Bussche H Hansmann-Wiest J Van Der Leeden C. The medical treatment of homeless people. Dtsch Arztebl Int. (2017) 114:673–9. 10.3238/arztebl.2017.0673 29070427 PMC5963585

