# Corrigendum: Increasing Number of Individuals Receiving Hepatitis B nucleos(t)ide Analogs Therapy in Germany, 2008–2019

**DOI:** 10.3389/fpubh.2022.860836

**Published:** 2022-03-11

**Authors:** Anna Maisa, Christian Kollan, Matthias an der Heiden, Florian van Bömmel, Markus Cornberg, Stefan Mauss, Heiner Wedemeyer, Daniel Schmidt, Sandra Dudareva

**Affiliations:** ^1^Department of Infectious Disease Epidemiology, Robert Koch Institute, Berlin, Germany; ^2^Division of Hepatology, Department of Medicine II, Leipzig University Medical Center, Leipzig, Germany; ^3^Department of Gastroenterology, Hepatology and Endocrinology, Hannover Medical School, Hannover, Germany; ^4^Center for HIV and Hepatogastroenterology, Düsseldorf, Germany; ^5^Charité Universitätsmedizin Berlin, Berlin, Germany

**Keywords:** hepatitis B, nucleos(t)ide therapy, therapy costs, treatment guidelines, treatment gap, hepatitis elimination

In the original article, there was a mistake in the order of figures for Figures 1–3 as published. Figure 1 should be Figure 3, Figure 2 should be Figure 1, and Figure 3 should be Figure 2. The legends and references in the manuscript for all three figures were correct. The corrected Figures 1–3 appear below.

**Figure 1:**
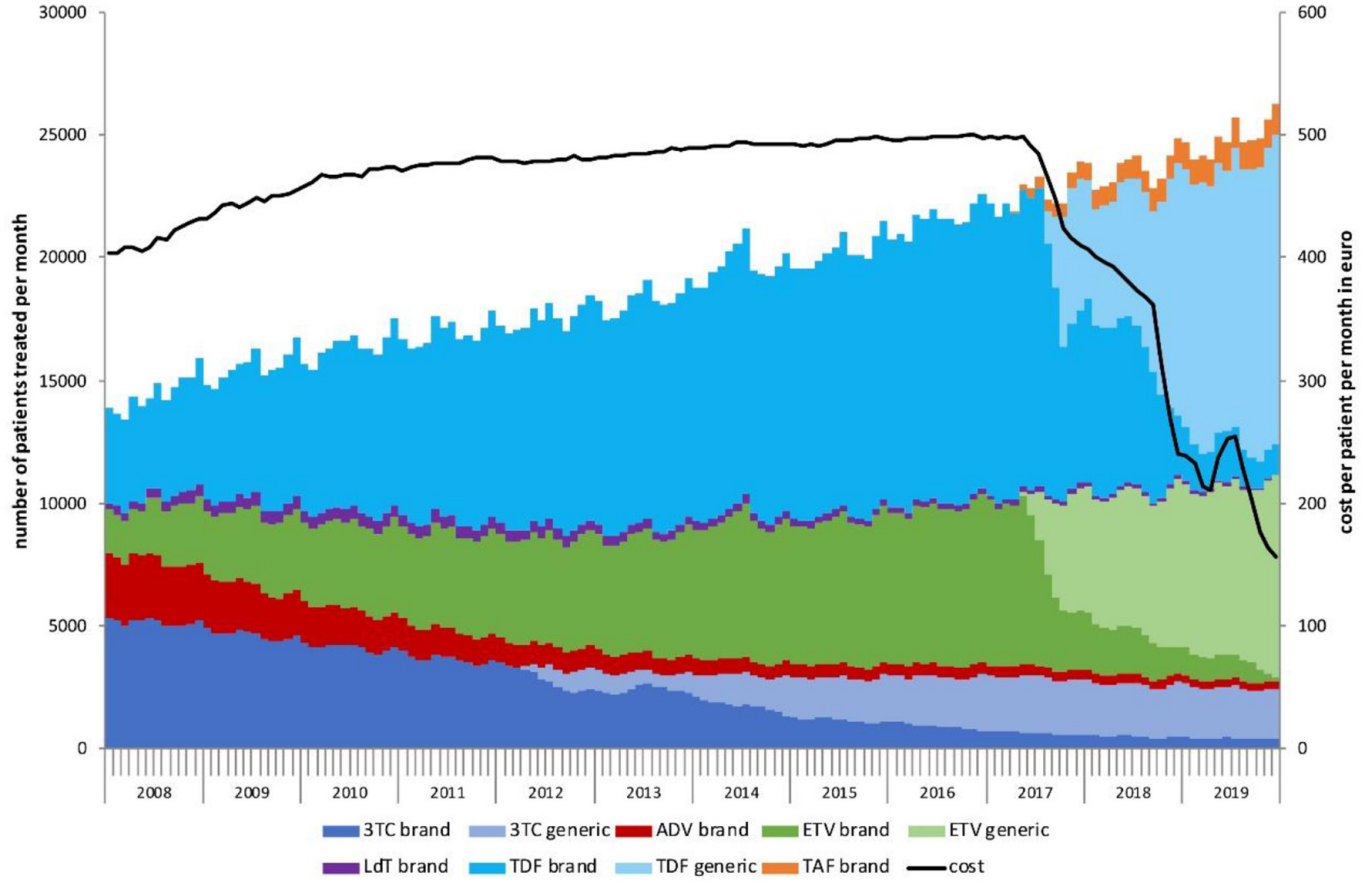
Number of chronic hepatitis B patients treated per month by drug, 2008–2019. The black line shows the cost per patient per month in Euro.

**Figure 2:**
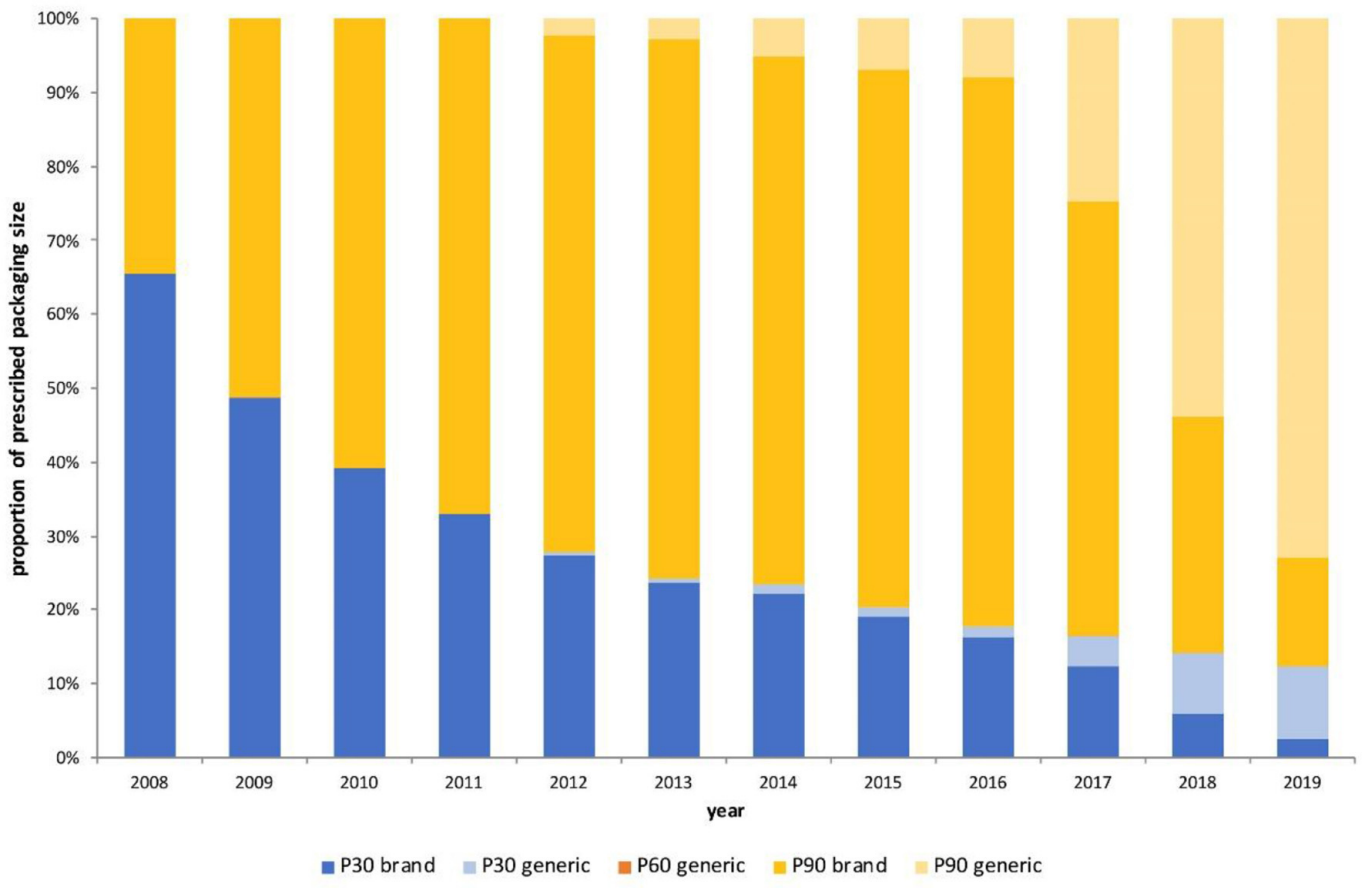
Proportion of prescriptions issued per year by prescription size, 2008–2019. P30–30 pills per prescription; P60–60 pills per prescription; P90–90 pills per prescription.

**Figure 3:**
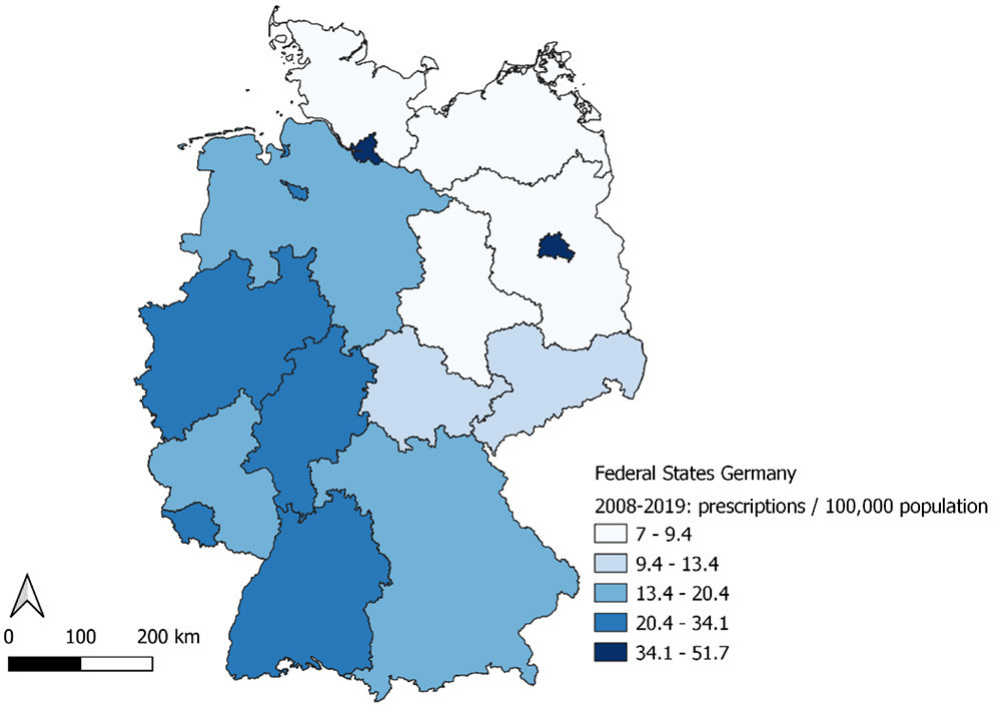
Prescriptions per 100,000 population by Federal State, Germany, 2008–2019.

The authors apologize for this error and state that this does not change the scientific conclusions of the article in any way. The original article has been updated.

## Publisher's Note

All claims expressed in this article are solely those of the authors and do not necessarily represent those of their affiliated organizations, or those of the publisher, the editors and the reviewers. Any product that may be evaluated in this article, or claim that may be made by its manufacturer, is not guaranteed or endorsed by the publisher.

