# Supplementary material for: Increasing Number of Individuals Receiving Hepatitis B nucleos(t)ide Analogs Therapy in Germany, 2008–2019
Source: Front Public Health. 2021 May 21;9:667253. doi: 10.3389/fpubh.2021.667253 (PMC8175796; doi:10.3389/fpubh.2021.667253)
Supplement: Supplementary file 1 [file Table_1.DOCX]

Supplementary Material

**Supplementary Table S1:** Annual average proportion of prescribed NUC drugs for HBV therapy, 2008 – 2019. Calculated from monthly proportions and rounded.

|  | 3TC |  | ADV | ETV |  | LdT | TDF |  | TAF |
| --- | --- | --- | --- | --- | --- | --- | --- | --- | --- |
|  | brand | generic | brand | brand | generic | brand | brand | generic | brand |
| 2008 | 36 | n/a | 18 | 15 | n/a | 2.8 | 29 | n/a | n/a |
| 2009 | 30 | n/a | 13 | 19 | n/a | 3.2 | 35 | n/a | n/a |
| 2010 | 25 | n/a | 9.4 | 21 | n/a | 3.1 | 41 | n/a | n/a |
| 2011 | 21 | n/a | 6.9 | 23 | n/a | 2.9 | 45 | n/a | n/a |
| 2012 | 16 | 3.1 | 5.2 | 25 | n/a | 2.5 | 49 | n/a | n/a |
| 2013 | 13 | 3.8 | 4.0 | 26 | n/a | 2.0 | 51 | n/a | n/a |
| 2014 | 8.8 | 6.3 | 3.1 | 29 | n/a | 1.6 | 52 | n/a | n/a |
| 2015 | 5.6 | 8.5 | 2.6 | 29 | n/a | 1.3 | 53 | n/a | n/a |
| 2016 | 4.2 | 9.4 | 2.2 | 30 | n/a | 1.1 | 53 | n/a | n/a |
| 2017 | 2.7 | 10 | 1.9 | 22 | 13 | 0.9 | 45 | 18 | 2.0 |
| 2018 | 2.1 | 9.0 | 1.6 | 7.4 | 24 | 0.7 | 25 | 27 | 3.6 |
| 2019 | 1.7 | 8.2 | 1.3 | 3.4 | 28 | 0.5 | 6.5 | 46 | 4.5 |

3TC – lamivudine; ADV – adefovir; ETV – entecavir; LdT – telbivudine; TDF – tenofovir disoproxil fumarate; TAF – tenofovir alafenamide; n/a – not available

**Supplementary Figure S2:** Cost per monthly units of prescribed NUC drugs for HBV therapy, 2008 – 2019. Negative binomial regression with expected trend using quadratic time function for better fit and 95% confidence interval.

**Supplementary Table S3:** Number of monthly units (MU) issued in January of each year, incidence rate ratio and percentage change (%) of MU costs of prescribed NUC drugs for HBV therapy, 2008 – 2019

| year | n | Incidence rate ratio | % |
| --- | --- | --- | --- |
| 2008 | 6089285 |  |  |
| 2009 | 6651243 | 1.092 | 9.2 |
| 2010 | 7222338.5 | 1.086 | 8.6 |
| 2011 | 7796350 | 1.079 | 7.9 |
| 2012 | 8366491 | 1.073 | 7.3 |
| 2013 | 8925526 | 1.067 | 6.7 |
| 2014 | 9465919 | 1.061 | 6.1 |
| 2015 | 9979994 | 1.054 | 5.4 |
| 2016 | 10460110 | 1.048 | 4.8 |
| 2017 | 10898851 | 1.042 | 4.2 |
| 2018 | 9338360 | 0.857 | -14 |
| 2019 | 6429236.5 | 0.688 | -31 |

**Supplementary Table S4:** Annual percentage change (%) of prescriptions of NUC drugs for HBV therapy by federal state, 2008 – 2019. Negative binomial regression with linear trend.

| Federal state | Percentage change per year |
| --- | --- |
| Berlin | 0.59 |
| Hamburg | 2.21 |
| Saxony | 3.55 |
| Thuringia* | 3.77 |
| Saxony-Anhalt | 4.88 |
| North Rhine-Westphalia | 5.32 |
| Hesse | 5.47 |
| Saarland | 5.53 |
| Baden-Wuerttemberg | 5.78 |
| Bavaria | 5.87 |
| Brandenburg | 5.95 |
| Schleswig-Holstein | 6.34 |
| Lower Saxony | 6.68 |
| Rhineland-Palatinate | 6.74 |
| Mecklenburg-Western Pomerania | 7.24 |
| Bremen | 7.97 |

*percentage increase calculated for 2014-2019
